# Hydrogel gratings with patterned analyte responsive dyes for spectroscopic sensing†

**DOI:** 10.1039/d1ra08610c

**Published:** 2021-12-17

**Authors:** Ruchi Gupta, Sameh El Sayed, Nicholas J. Goddard

**Affiliations:** School of Chemistry, University of Birmingham Birmingham B15 2TT UK s.elsayed@bham.ac.uk r.gupta.3@bham.ac.uk; Process Instruments (UK) Ltd Turf Street Burnley BB11 3BP UK nick.goddard@processinstruments.net

## Abstract

This is an unprecedented report of hydrogel gratings with an analyte responsive dye immobilised in alternating strips where the patterned dye is its own dispersive element to perform spectroscopy. At each wavelength, the diffraction efficiency of hydrogel gratings is a function of dye absorbance, which in turn is dependent on the concentration of analytes in samples. Thus, changes in intensity of diffracted light of hydrogel gratings were measured for sensing of analytes. Equally, the ratio of diffracted intensities at two wavelengths was used for quantification of analytes to reduce errors caused by variations in intensity of light sources and photobleaching of dyes. 15.27 μm pitch gratings were fabricated by exposing 175 μm thick films of photofunctionalisable poly(acrylamide) hydrogel in a laser interferometric lithography setup, generating an array of alternating lines with and without free functional groups. The freed functional groups were reacted with pH sensitive fluorescein isothiocyanate to create gratings for measurement of pH. The ratio of intensity of diffracted light of hydrogel gratings at 430 and 475 nm was shown to be linear over 4 pH units, which compares favourably with ∼2 pH units for conventional absorption spectroscopy. This increased dynamic range was a result of cancellation of the opposite non-linearities in the pH response of the analyte responsive dye and the diffraction efficiency as a function of dye absorbance.

## Abstract 2

This is an unprecedented report of hydrogel gratings with an analyte responsive dye immobilised in alternating strips where the patterned dye is its own dispersive element to perform spectroscopic sensing.

## Graphical abstract

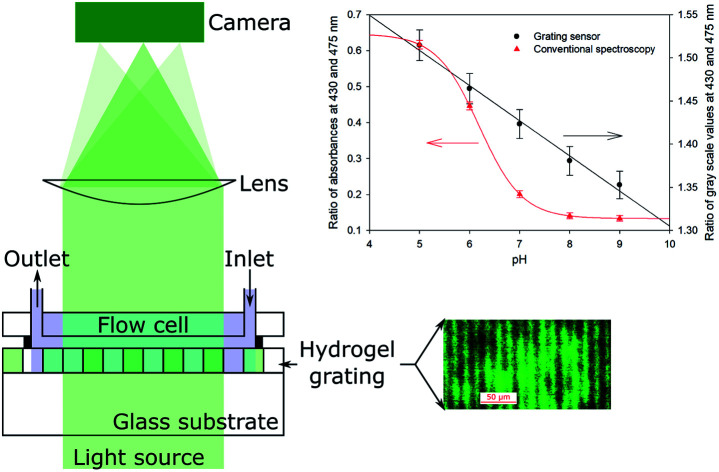

## 1. Introduction

Spectroscopic sensing is ubiquitous for clinical diagnostics^1,2^ and environmental monitoring.^3,4^ The principle of spectroscopic sensing is to measure analytes by recording their optical transmission or reflection *versus* wavelength. Spectroscopic sensing is beneficial over single wavelength studies because it can allow measurement of multiple analytes without separation^5^ and ratiometric analysis to reduce errors.^6,7^ Many analytes do not absorb in the visible region and hence are measured indirectly by recording changes in optical spectra of probe species. These changes in optical spectra of probe species are caused by their interactions with analytes. Such analyte responsive probes can be organic dyes,^8,9^ lanthanides,^10^ and functional nanomaterials^11^ such as nanoparticles and quantum dots. For example, the concentration of hydrogen ions, *i.e.* pH, can be measured by recording changes in the absorption spectrum of an organic dye, fluorescein, which has an isosbestic point at ∼460 nm, p*K*_a_ of ∼6.5, and changes its absorbance at 490 nm between pH 5 and pH 9.^12^ Equally, analyte responsive dyes have been attached to solid and porous matrices and their wavelength-dependent optical transmission has been measured over time to extend the applicability of spectroscopic sensing for continuous measurement of analyte concentrations.^13^

Spectroscopic sensing requires that light is dispersed into its wavelength components, which is achieved using gratings that are separate from the matrices with analyte responsive dyes. This work, for the first time, integrates these two essential components of spectroscopic sensors by developing hydrogel gratings comprising an array of lines without and with analyte responsive dyes. Such hydrogel gratings with patterned analyte responsive dye allowed both sensing and dispersing of white light to perform spectroscopy. The sensing principle of the reported hydrogel gratings is that their diffraction efficiency is dependent on analyte concentrations because analyte concentrations modulated dye absorbance. To demonstrate spectroscopic sensing, gratings with patterned fluorescein were fabricated and used for measurement of an exemplar analyte, concentration of hydrogen ions, which is related to the pH of sample solutions. The relationship between intensity of diffracted light of fluorescein patterned hydrogel gratings and pH was linear over 4 pH units, which is better than conventional absorption spectroscopy.

Hydrogel gratings for sensing applications have previously been made by soft lithography^14^ and exposure of photopolymers^15^ or gelatin-silver halide emulsions^16^ to light patterns. Previous work on hydrogel gratings has only produced low aspect ratio structures (<1), because all previous methods at some stage require free-standing hydrogel strips which do not have the required rigidity to be self-supporting at high aspect ratios.^17^ In this work, high aspect (∼23) ratio patterns were produced by photodeprotection of amines in a hydrogel of acrylamide and copolymer. Subsequently, the patterned free amines were reacted with an analyte responsive dye to form a grating. In this case, the dye was its own dispersive element, replacing an external grating or prism. Photofunctionalisable hydrogels have so far been primarily used for tissue engineering and drug delivery applications^18^ with an exception being the work done by authors where photofunctionalisable hydrogels were used to generate gratings.^19^ However, previously reported hydrogel gratings were not suitable for spectroscopic sensing because their pitch was ≥43.2 μm,^19^ resulting in insufficient angular separation between zero and first diffracted orders when the gratings were illuminated with white light. In this work, analyte responsive gratings with a pitch of 15.27 μm were fabricated in 175 μm thick films of photofunctionalisable hydrogels, and their suitability for spectroscopic sensing was demonstrated by measuring an exemplar analyte, pH.

## 2. Experimental

### Chemicals and materials

Chemicals required to synthesise photolabile monomer included 4,5-dimethoxy-2-nitrobenzyl chloroformate (NVOC), methacrylamide poly(ethylene glycol) amine hydrochloride (*M*_n_: 400), triethylamine (TEA), anhydrous tetrahydrofuran (THF), ethyl acetate, hydrochloric acid (HCl), and sodium sulphate (Na_2_SO_4_). These chemicals were bought from Sigma-Aldrich.

Ethanol, chloro(dimethyl)vinyl silane (CDVS), toluene, 40% (w/v) solution of 29 : 1 (w/w) acrylamide : bis-acrylamide, ammonium persulphate (APS), *N*,*N*,*N*′,*N*′-tetramethylethylenediamine (TEMED), sodium phosphate monobasic monohydrate, sodium phosphate dibasic dodecahydrate, sodium phosphate tribasic dodecahydrate, 1 M sodium hydroxide, hydroxylamine (NH_2_OH) hydrochloride, and fluorescein isothiocyanate (FITC) were bought from Sigma-Aldrich. Microscope glass slides were bought from VWR. These materials and chemicals were used to fabricate photofunctionalisable hydrogel films on glass substrates.

Materials used to make fluidic flow cells consisted of 3 mm thick transparent poly(methyl methacrylate) (PMMA) sheets, bootlace ferrules (211-4252), and nitrile O-rings (01-08-01801), and were purchased from Fred Aldous, RS Components, and Ashton Seals, respectively.

### Synthesis of photolabile monomer

Methacrylamide poly(ethylene glycol) amine hydrochloride (392 mg, 0.71 mmol) and TEA (184 mg, 1.81 mmol) were dissolved in a round bottom flask containing anhydrous THF (10 ml) under inert atmosphere in ice bath for 30 min. Then a solution of a widely used photoprotecting compound,^20,21^ NVOC (250 mg, 0.91 mmol) was dissolved in anhydrous THF and under dark conditions at 0 °C was added dropwise to the first solution over 30 min. The resulting mixture was stirred at this temperature for 2 h and then at room temperature overnight. THF was evaporated by N_2_ gas flow, then 5 ml of distilled water was added, and the mixture was extracted with ethyl acetate (2 × 15 ml). The aqueous layer was collected, acidified (pH 4.0) by addition of 2 ml of 5% (v/v) HCl at 0 °C, and extracted with cold ethyl acetate (3 × 15 ml). The collected extract was dried over Na_2_SO_4_ and concentrated at reduced pressure for 24 h at 0 °C. The photolabile monomer was stored under dark conditions in a fridge at 4 °C (yield 89%). The formation of the photolabile monomer was verified by the NMR spectroscopy (see Fig. SI1 in ESI†).

### Fabrication of hydrogel gratings

Glass slides were cut into ∼25.4 × 25.4 mm^2^ squares, and cleaned in Decon 90, de-ionised water and ethanol for 20 minutes each. Glass squares were immersed in 0.5% (v/v) CDVS in toluene for 20 min, washed in toluene, and dried before use. Thus, glass squares were functionalised with vinyl groups. These vinyl groups can undergo free radical polymerisation with hydrogel precursor solutions, allowing covalent attachment between glass squares and hydrogel films.

1 ml of hydrogel precursor solution containing 5% (w/v) of total monomer, 27 mM photolabile monomer and 540 mM acrylamide, 5 μl of TEMED and 50 μl of 10% (w/v) APS was prepared in N_2_-degassed water. The molar ratio of photolabile monomer to acrylamide was 5%. The precursor solution was cast between CDVS treated glass squares and plastic covers separated with 175 μm spacers (939-837-76, Goodfellow) at room temperature under dark. Typical polymerisation time for precursor solutions was <10 min. After the solution polymerised, the plastic cover was removed, and hydrogel films deposited on glass substrates were stored in 100 mM phosphate buffer, pH 8.0 and in dark.

Unless stated otherwise, all solutions were prepared in 100 mM phosphate buffer, pH 8.0. Hydrogel films were immersed in 20 ml of freshly prepared 12.5 mg ml^−1^ NH_2_OH solution (pH adjusted to 8.0) for 30 min. To fabricate hydrogel gratings, films were exposed to an array of bright and dark fringes produced by the interference of two beams in a laser interference lithography (IL) set-up. The photolabile monomer was deprotected in regions of films exposed to bright lines, and hence films comprising an array of alternative lines with free and caged amines were obtained. Patterned films were washed in buffer overnight in darkness, immersed in 0.1 mg ml^−1^ of FITC solution for at least 1 h, and washed in buffer again. The resulting films comprised an array of lines with and without immobilised FITC and served as amplitude gratings. The amplitude gratings were stored in buffer and in dark until use.

### Instrumentation

The details of key instrumentation used in this work are provided below.

### Laser interference lithography (IL) set-up

**Fig. 1:**
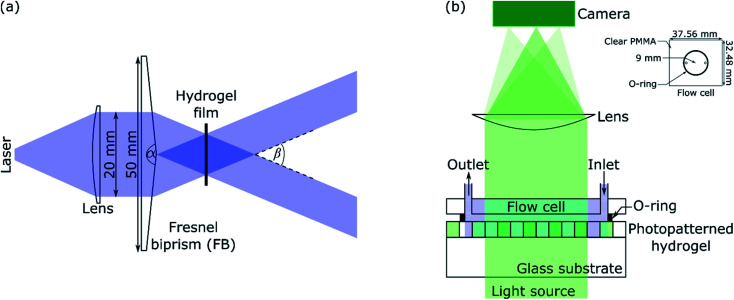
(a) Laser IL set-up (*α* and *β* are apex and crossing angles, respectively) and (b) set-up for diffraction studies where inset shows the dimensions of the flow cell.

A schematic of the IL set-up used in this work is shown in Fig. 1(a). Light from a laser (L375P70MLD Thorlabs) with a peak wavelength (*λ*_IL_) of 375 nm and power of 70 mW was passed through a collimating lens (63DQ25, Comar Optics) to obtain a beam with a diameter of 20 mm. The power density of the expanded laser beam was ∼22 mW cm^−2^. The beam was split into two by passing through a Fresnel biprism (FB) with an apex angle (*α*) of either 179 deg (3B Scientific) or 177 deg (Newlight Photonics). The two beams interfered to produce an array of bright and dark fringes. These fringes were projected on hydrogel films immersed in NH_2_OH to selectively deprotect the photolabile monomer in regions exposed to bright lines.

### Set-up for diffraction studies

A green or red laser (CW532-005, LDM650/3LJ, both: Roithner Lasertechnik) or white LED (Luxeon LXZ1-2780-y 2700K, RS Components) was used to perform diffraction studies. Light from the LED was collimated by passing through an achromatic doublet (63DQ25, Comar Optics). The beam was passed through an aperture of 5 × 8 mm^2^, and then as shown in Fig. 1(b), illuminated hydrogel gratings at 0 deg angle of incidence. Transmitted light was passed through a cylindrical lens (160YE25, Comar Optics) and captured on a camera (Mercury Daheng MER-2000-19U3M, GeT Cameras BV) to obtain diffraction images. The camera had 5496 by 3672 pixels with each pixel being 2.4 × 2.4 μm. As shown in Fig. 1(b), a flow cell was made by sandwiching an O-ring between a PMMA sheet with through holes and hydrogel film. The O-ring had an internal radius of 9 mm and thickness of 1 mm. As shown in the inset in Fig. 1(b), the PMMA sheet was 32.48 mm wide and 37.56 mm long. The diameter of each through hole was 1.35 mm, and their centre-to-centre distance was 13 mm. Bootlace ferrules were glued in the through holes to make fluidic inlets/outlets. The inlet of the flow cell was connected to a MINIPULS 3 peristaltic pump (Gilson) and outlet tubing was placed in a waste beaker. Solutions were pumped at a volumetric flow rate of 3 μl s^−1^.

The pH and absorption spectra of solutions were measured using HI-2210-02 Bench Top pH Meter (Hanna Instruments) and Jenway 6715 UV-Vis spectrometer, respectively. Fluorescence images were taken using Axio Imager M2 connected to Axiocam 503 camera (1936 by 1460 pixels with each pixel being 4.54 × 4.5 μm, both: Zeiss) and analysed using Fiji – Image J.

## 3. Results and discussion

### Characterisation of monomer and hydrogels

Based on eqn SI1 and SI2 in ESI,† the molar extinction coefficients of the photolabile monomer at 350 and 375 nm (*ε*_350_ and *ε*_375_) were 5773 ± 39 M^−1^ cm^−1^ and 4200 ± 6 M^−1^ cm^−1^, respectively. *ε*_350_ of the synthesised photolabile monomer is slightly higher than literature reported values of 5100 to 5484 M^−1^ cm^−1^, but this may reflect different solvents used for the measurements. The absorbance of freshly prepared 175 μm thick photofunctionalisable hydrogel films at 350 nm was 0.197. Thus, the concentration of the photolabile monomer in hydrogels was estimated to be 1.9 mM. This implies that the molar ratio of the photolabile monomer to acrylamide in hydrogel films was <1%.

To determine photolysis kinetics, photofunctionalisable hydrogel films were immersed in solutions of NH_2_OH scavenger^22^ and exposed to 375 nm peak wavelength laser beam. The time at which peak absorbance of NVOC in films was dropped to half of the initial value was 6.91 ± 0.24 min, which is in agreement with literature^23^ after accounting for laser peak wavelength and power density. Free amines generated in photofunctionalisable hydrogels after exposure to the laser beam for 10 to 40 min were reacted with FITC. In all cases, the peak absorbance of films with immobilised FITC was ∼0.236 at pH 8.

Control experiments were undertaken to show that the photolabile monomer was incorporated into the hydrogel film and was photolabile. Polyacrylamide films without the photolabile monomer showed no reactivity to FITC, remaining uncoloured. Similarly, films containing the photolabile monomer that were not exposed to UV did not react with FITC. Only films containing the photolabile monomer and exposed to UV reacted with FITC. The reaction of FITC with freed amines was confirmed by measuring UV-Vis absorption spectra of hydrogel films.

### Pitch of hydrogel gratings

**Fig. 2:**
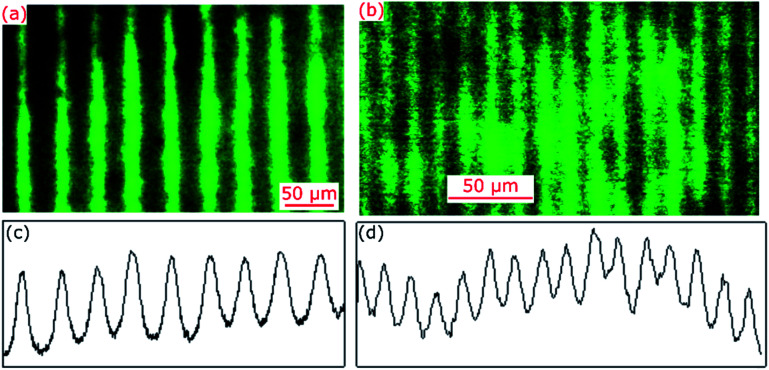
Fluorescence microscope images and corresponding cross-sectional profiles of hydrogel films patterned using the IL set-up comprising FB with *α* of (a and c) 179 deg and (b and d) 177 deg.

Hydrogel films comprising an array of lines without and with FITC were imaged using a fluorescence microscope and exemplar images are provided in Fig. 2(a) and (b). The corresponding cross-sectional profiles obtained by vertical averaging over the entire image (see Fig. 2(c) and (d)) show that fluorescence intensity and hence distribution profile of immobilised FITC was cosinusoidal in patterned hydrogel films. This is to be expected because light intensity of interference fringes of the laser IL set-up is cosinusoidal.^24^ The cross-sectional profiles shown in Fig. 2(c) and (d) were fitted to cosinusoidal curves to determine observed pitch of arrays of lines (*Λ*_obs_). As listed in Table 1, *Λ*_obs_ were 40.13 ± 0.04 and 15.27 ± 0.02 μm for hydrogel gratings fabricated using the FB with *α* of 179 deg and 177 deg, respectively.

**Table 1:** Summary of expected and observed pitch of arrays of lines patterned in hydrogels

	*α* (deg)	*n*_FB_ (*λ*_IL_: 375 nm)	*β* (deg)	*Λ*_exp_ (μm)	*Λ*_obs_ (μm)
FB1	179	1.5345	0.5345	40.20	40.13 ± 0.04
FB2	177	1.4731	1.4199	15.10	15.27 ± 0.02

The expected pitch (*Λ*_exp_) of hydrogel gratings obtained by exposure to the laser IL set-up with a FB is given by eqn (1) where the relationship between *α* and beam crossing angle (*β*) is given by eqn (2). *n*_FB_ is refractive index of FB and *λ*_IL_ is the wavelength of the laser used in the IL set-up *i.e.*, 375 nm.1
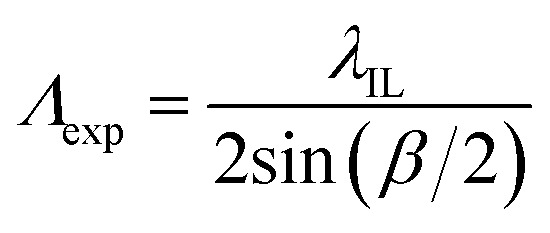

2 *β* = *α* − 2 cos^−1^(*n*_FB_ cos(*α*/2))

A comparison of *Λ*_obs_ and *Λ*_exp_ in Table 1 suggests that observed pitch of arrays of lines was <1.1% different from values expected based on theory. For hydrogel films patterned with arrays of lines with a pitch of 15.27 ± 0.02 μm, the aspect ratio of each strip to film thickness was ∼23.

### Diffraction studies

**Fig. 3:**
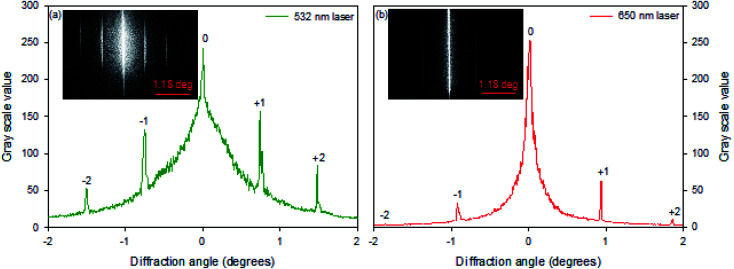
Diffraction patterns 40.13 μm pitch hydrogel gratings illuminated with lasers of peak wavelength of (a) 532 nm and (b) 650 nm.

Hydrogel gratings were illuminated with 532 and 650 nm lasers and diffraction patterns were obtained in both cases as shown in Fig. 3. FITC absorbs between 400 and 540 nm, and hence pure amplitude gratings with alternate lines of FITC will only result in diffraction patterns when illuminated with 532 nm light. However, as a diffraction pattern was also observed when gratings were illuminated with 650 nm laser (Fig. 3(b)), hydrogel gratings are combined amplitude-phase gratings. This is to be expected because it is unlikely that adjacent lines with FITC (but no NVOC) and NVOC (but no FITC) will have the same real refractive index.

### Design of gratings

Pitches of gratings fabricated using both FBs were substituted in eqn SI3† and the grating parameter,^25^*Q*, was not much greater than one, which means that gratings described here are all thin. The fluorescence microscope images of fabricated gratings indicated that distribution of immobilised FITC was cosinusoidal. Furthermore, diffraction studies using 532 and 650 nm lasers suggested that fabricated hydrogel gratings are combined amplitude-phase gratings. Thus, theory developed by Song *et. al*.^26^ was adapted (see ESI† for details) to determine theoretical diffraction efficiency and design gratings with optimal sensing performance.

Eqn SI4† in the ESI† gives the change in the diffraction efficiency as a function of the maximum absorbance of the dye strips, which shows that the maximum sensitivity is reached when the absorbance is 0.301. The exposure time of the photofunctionalisable hydrogel was optimized at 10 min to obtain an absorbance of 0.236 at a pH of 8, which is close to the absorbance for maximum sensitivity. Longer exposures in the laser interferometry setup did not significantly increase the absorbance to reach the theoretical maximum sensitivity.

The grating pitch was controlled by the apex angle of the Fresnel biprism and wavelength of the laser. Gratings produced with the 177 deg FB had a pitch of 15.27 μm, and were used for pH sensing using white light, as these are expected to give an angular separation of 1.78, 2.00 and 2.44 deg for 475, 532 and 650 nm light, respectively. As the focal length of the cylindrical lens between the grating and camera was 160 mm, the separation between the zero and first orders was 4.98 mm at 475 nm. This resulted in a separation of over 2000 pixels between the zeroth and first orders. As the zeroth and first diffracted orders of 15.27 μm pitch gratings were well separated, finer pitch gratings were not investigated.

### Spectroscopic sensing

**Fig. 4:**
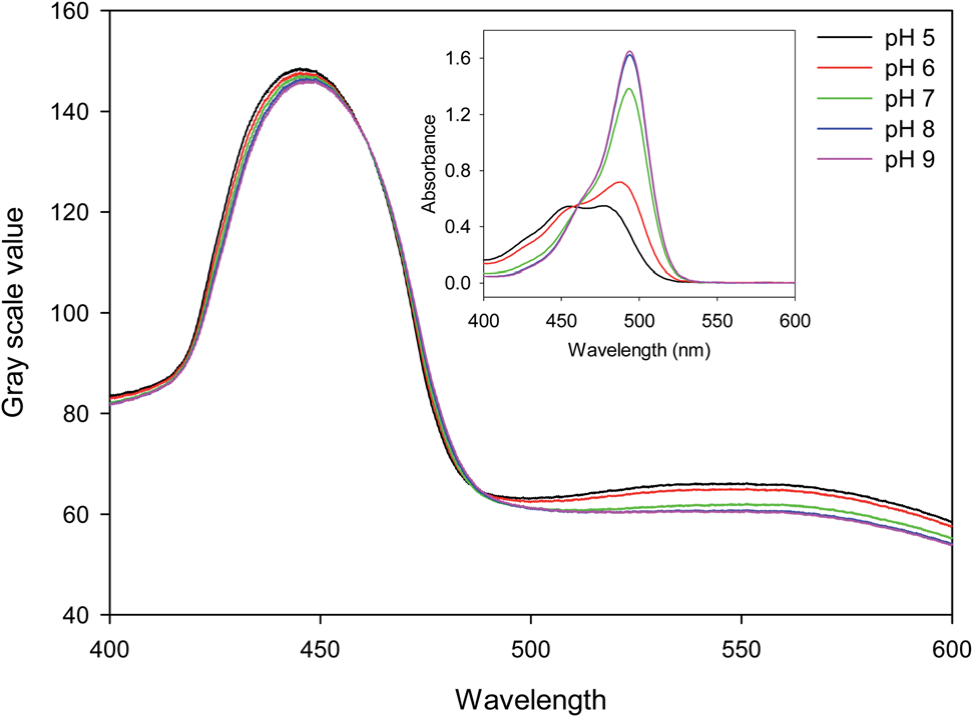
Gray scale value of first diffracted order *versus* wavelength when hydrogel gratings are irrigated with 10 mM phosphate buffer solutions of different pH where inset shows absorption spectrum of 5 μg ml^−1^ fluorescein solutions of different pH in 1 cm pathlength cuvettes.

15.27 μm pitch hydrogel gratings formed by patterning of FITC were illuminated at normal incidence with white light and transmitted light was recorded using a camera to obtain their diffraction patterns. A typical plot of gray scale value *versus* wavelength of first diffracted order is provided in Fig. 4 where interference filters of known peak wavelength were placed in the path of white light to determine the relationship between distance (camera pixels) and wavelength (nm). While the upper envelope of the gray scale values was determined by the spectral intensity distribution of the white LED, gray scale value *versus* wavelength was directly proportional to diffraction efficiency of hydrogel gratings at each wavelength. Furthermore, based on eqn SI4 in ESI,† diffraction efficiency of first diffracted orders is a function of dye (in this case, fluorescein) absorbance. Inset in Fig. 4 shows that fluorescein has an isosbestic point at ∼460 nm where absorption is independent of pH. As pH increases from 5 to 9, absorption of fluorescein decreases in wavelength range 400–455 nm, but increases in wavelength range 465–520 nm. In Fig. 4, since changes in diffraction efficiency with pH were observed at wavelengths above 540 nm where fluorescein does not absorb, it appears that we have a combined amplitude-phase grating at wavelengths below 540 nm and a pure phase grating at wavelengths above 540 nm. It should be noted, however, that the changes in diffraction efficiency at wavelengths below 540 nm cannot be a result only of refractive index change as there is no difference in diffraction efficiency at pH 8 and 9 at wavelengths above 540 nm whereas there is a difference below 540 nm. In addition, since changes in diffraction efficiency at wavelengths on either side of the isosbestic point are in opposite directions, the observed changes below 540 nm must be mainly because of absorbance change.

**Fig. 5:**
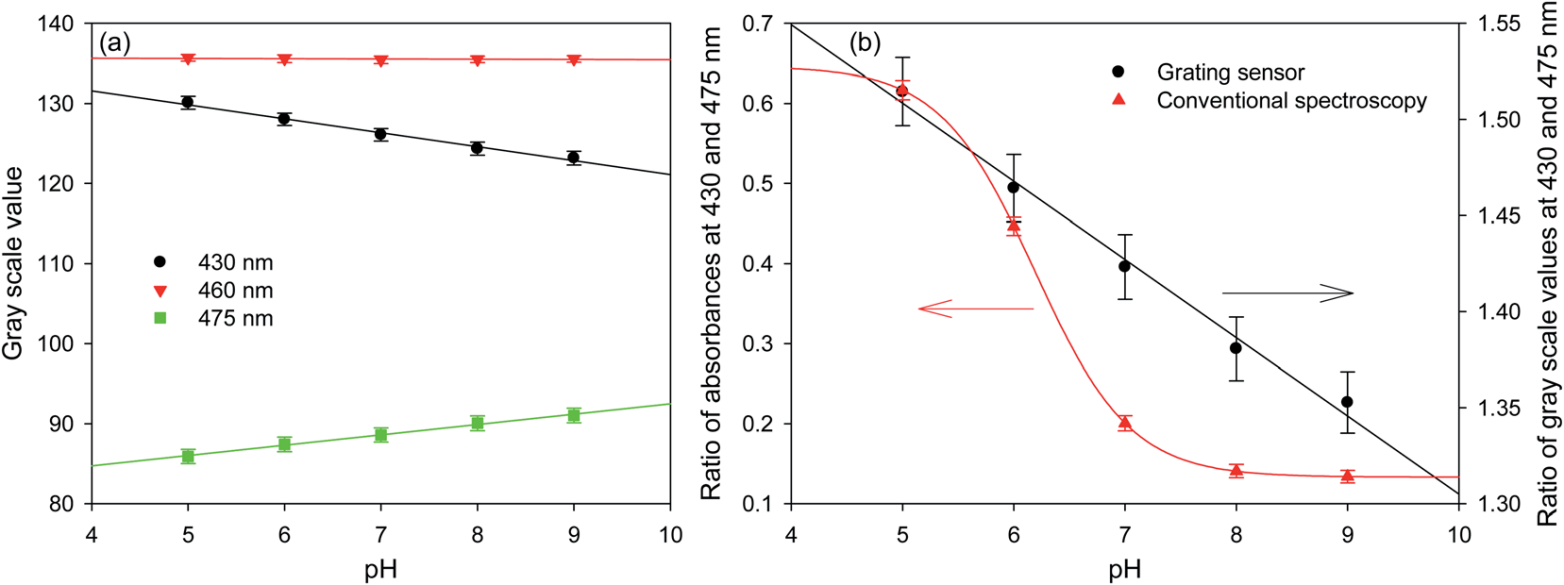
Plots of (a) gray scale values of diffracted orders at three wavelengths and (b) ratio of gray scale values and absorbances at 430 and 475 nm for hydrogel gratings and conventional spectroscopy, respectively, as a function of pH of solutions (error bars are ±1 standard deviation derived from the intensity variations around the wavelengths of interest).

Fig. 5(a) shows that the gray scale value of first diffracted order of gratings is ∼135 at isosbestic wavelength of FITC *i.e.*, at 460 nm. The relationships between gray scale values of first diffracted orders at 430 and 475 nm (GS_430_ and GS_475, respectively_) was linear with pH, and are given by eqn (3) and (4), respectively. Thus, measurement sensitivity of hydrogel gratings at 430 and 475 nm was −1.75 and 1.29 per pH unit, respectively.

3 GS_430_ = 138.61 − 1.75 pH, *r*^2^: 0.9902

4 GS_475_ = 79.55 + 1.29 pH, *r*^2^: 0.9956

Although changes in gray values of first diffracted orders at either 430 or 475 nm can be monitored to determine pH of solutions, this approach is prone to errors caused by fluctuations in the intensity of light sources and photobleaching of dyes patterned in hydrogels to make gratings. These errors can be reduced by performing ratiometric measurements between gray scale value of first diffracted orders at 430 and 475 nm. Using the ratio of gray scale values of the first diffracted order at 430 and 475 nm gives greater sensitivity than the ratios between values at 430 or 475 and the isosbestic point at 460 nm. As shown in Fig. 5(b), relationship between GS_430_/GS_475_ and pH was linear, and is given by eqn (5).5
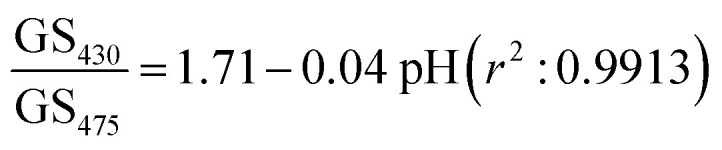


Fig. 5(b) shows that the hydrogel grating sensor gives a linear ratiometric response, but the conventional spectrophotometric ratiometric response is, as expected, sigmoidal. This means that the dynamic range of the grating sensor is about 4 pH units compared to about 2 pH units for the conventional measurement.

The wider dynamic range obtained from the grating sensor is a result of the non-linearity of the absorbance of fluorescein as a function of pH being largely cancelled out by the opposite non-linearity of the diffraction efficiency as a function of absorbance. As the absorbance change for fluorescein at 475 nm decreases at higher pH, this is compensated by the increased diffraction efficiency, resulting in a linearised response to pH over 4 pH units. The same effect is observed at 430 nm.

**Fig. 6:**
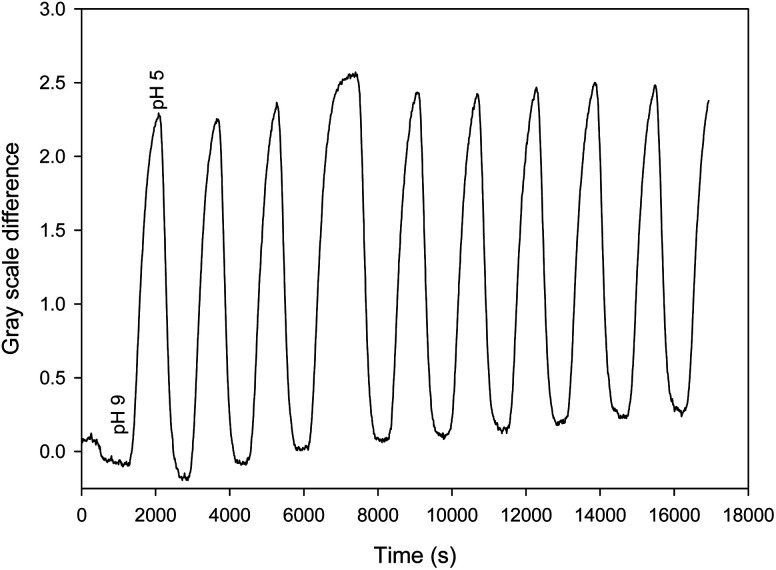
Plot of gray scale difference around 430 and 475 nm as a function of time as the pH was alternated between 5 and 9.

Finally, the device was shown to be capable of repeatable real-time monitoring of pH for nine cycles of pH between 5 and 9 over five hours. The measurement was performed by determining the gray scale difference between the intensities around 430 and 475 nm to remove drift. Fig. 6 shows the resulting plot of gray scale difference as a function of time as the pH was alternated between 5 and 9 over a period of 5 hours.

## 4. Conclusions

This work has shown that gratings formed from analyte responsive dyes can provide spectroscopic information without the use of external spectrophotometers. The spectroscopic information allows ratiometric measurements to be made, eliminating common sources of errors including photobleaching and fluctuations in light intensity. Gratings were fabricated by selective exposure of photofunctionalisable hydrogels to light for generating spatial patterns of free functional groups, which were then reacted with an analyte responsive dye. The exemplar analyte was pH and the exemplar analyte responsive dye was fluorescein. The ratio of gray scale values of the first diffracted orders at 430 and 475 nm was shown to be linear with pH over 4 pH units. This contrasts with conventional spectroscopy, which resulted in a sigmodal response and smaller dynamic range for pH measurements. The linearity of response is a consequence of the non-linearities of the absorbance as a function of pH and grating diffraction efficiency as a function of absorbance largely cancelling out, and distinguishes this form of sensor from conventional spectroscopy. In principle, any analyte responsive dye or functional nanomaterial can be used in the same way to realise hydrogel gratings for spectroscopic sensing.

## Author contributions

RG secured funding and supervised the work. RG and NJG conceptualised the work, analysed data, and prepared manuscript drafts. All authors contributed to experiments and proofread manuscript drafts.

## Conflicts of interest

Authors declare no conflict of interest.

## Supplementary Material

RA-011-D1RA08610C-s001
