# Supplementary material for: Hydrogel gratings with patterned analyte responsive dyes for spectroscopic sensing
Source: RSC Adv. 2021 Dec 17;11(63):40197–204. doi: 10.1039/d1ra08610c (PMC9044543; doi:10.1039/d1ra08610c)
Supplement: RA-011-D1RA08610C-s001 [file RA-011-D1RA08610C-s001.pdf]

Electronic Supplementary Material (ESI) for RSC Advances.
This journal is © The Royal Society of Chemistry 2021

Hydrogel Gratings with Patterned Analyte Responsive Dyes for Spectroscopic Sensing

Corresponding author: Ruchi Gupta, School of Chemistry, University of Birmingham, Birmingham, B15 2TT, UK; Email: r.gupta.3@bham.ac.uk.

Sameh El Sayed, School of Chemistry, University of Birmingham, Birmingham, B15 2TT, UK; Email: s.elsayed@bham.ac.uk

Nicholas J. Goddard, Process Instruments (UK) Ltd, Turf Street, Burnley, BB11 3BP, UK; Email: nick.goddard@processinstruments.net.

The formation of the photolabile monomer was verified by the NMR spectroscopy. 1H NMR (Bruker 300 MHz, DMSO-d6) δ 7.95 (s, 1H), 7.67 (d, J = 4.6 Hz, 1H), 7.36 (s, 1H), 5.65 (t, J = 1.3 Hz, 1H), 5.32 (t, J = 1.6 Hz, 1H), 5.03 (s, 2H), 4.03 – 3.70 (m, 10H), 3.61 – 3.39 (m, 29H), 3.32 – 3.20 (m, 2H), 3.04 (q, J = 7.3 Hz, 9H), 1.84 (dd, J = 1.6, 0.9 Hz, 3H). As shown in Figure SI1, the peak of two benzyl proton in NVOC reactant shifted from 513 for chloroformate to 5.03 of carbamate form (marked with *). The molecular weight of the photolabile monomer was 763.82 g mol^{-1}.

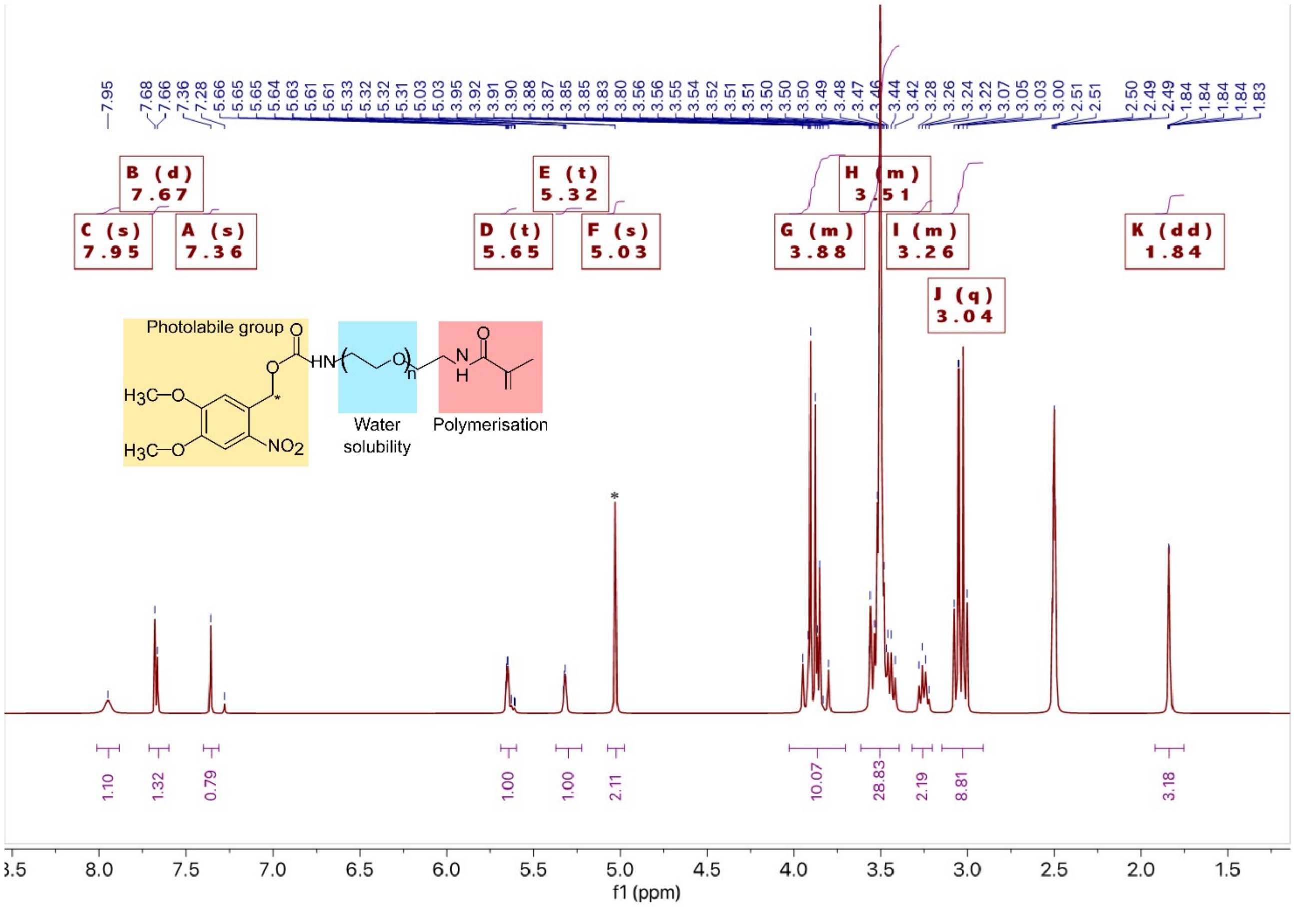

Figure SI1: NMR spectra of the photolabile monomer synthesised in this work where inset shows chemical structure of the monomer

Figure SI2 shows that the peak absorbance wavelength of the photolabile monomer was 350 nm, which is consistent with the values reported in literature[1]. As shown in inset in Figure SI2, the relationship between

absorbance (A_λ) and concentration of the photolabile monomer ($c_{monomer}$) in mM at 350 and 375 nm was linear at ≤0.26 mM, and is given by Equations SI1 and SI2, respectively.

$$A_{350} = 0.06 + 5.77c_{monomer} \qquad (r^2\text{: } 0.99996) \qquad \text{(SI1)}$$

$$A_{375} = 0.04 + 4.20c_{monomer} \qquad (r^2\text{: } 0.999998) \qquad \text{(SI2)}$$

As the monomer comprised of hydrophobic photolabile group and hydrophilic poly(ethylene glycol), it may form micelles, causing the slope of the absorbance curve in inset in Figure SI2 to change at monomer concentrations >0.26 mM. The monomer concentration at which deviation in the slope of the absorbance curve is observed is comparable to typical critical micellar concentrations reported in literature[2, 3].

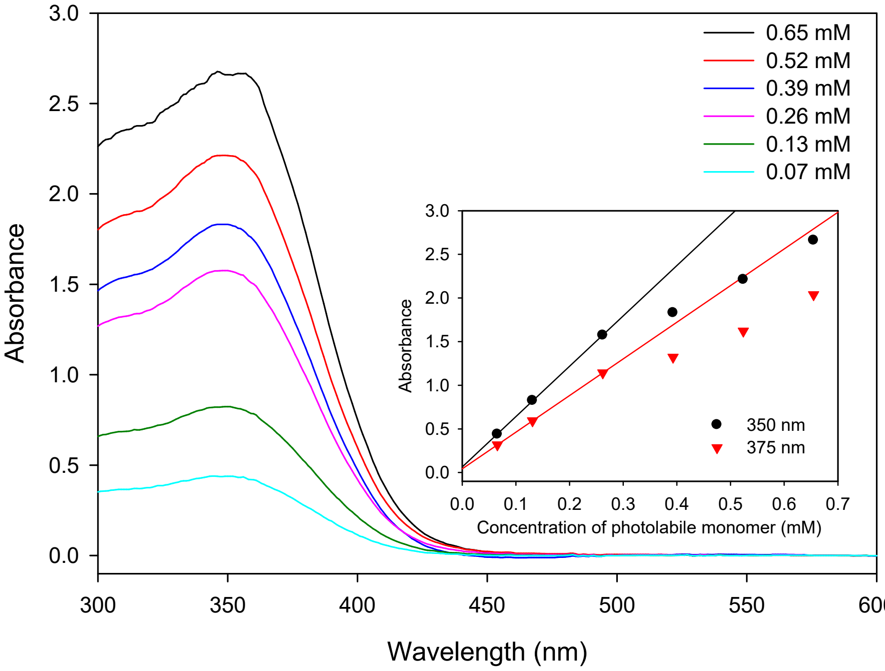

Figure SI2: Absorbance spectra of the photolabile monomer in water where inset shows absorbance at 350 and 375 nm *versus* monomer concentration

Grating theory. Gratings can be classified as thin or thick, depending on their geometrical thickness relative to pitch and wavelength of light. Towards this end, Kogelnik[4] defined a parameter Q, which is given by Equation SI3 where n, d and Λ are average refractive index, thickness and pitch of grating strips, respectively, and λ is wavelength of light used.

$$Q = \frac{2\pi\lambda d}{n\Lambda^2} \qquad \text{(SI3)}$$

At λ of 490 nm, an average n of 1.34 and d of 175 µm, the calculated Q based on the theoretical grating pitches is 0.249 for the hydrogel gratings produced by the 179° FB and 1.76 for the 177° FB. For a thick grating, the

condition $Q \gg 1$ must be satisfied[4]. The gratings described here do not satisfy this condition, so were considered thin for subsequent analysis.

Song *et. al.*[5] showed that the diffraction efficiency of thin cosinusoidal amplitude-phase gratings can be generated from the convolution of the diffracted amplitudes of the separate amplitude and phase components of the grating. The diffraction efficiency (η_1) of first diffracted orders is given by Equation SI4.

$$\eta_1 = |A_1|^2 = \frac{\left(1 - 10^{-A_a}\right)^2}{16}\left[J_0(\varphi_1) - J_2(\varphi_1)^2\right]^2 + \frac{\left(1 + 10^{-A_a}\right)^2}{4}\left[J_1(\varphi_1)\right]^2$$

(SI4)

Where A_1 is amplitude of first diffracted order, A_a is the maximum absorbance of the dye strips, $\varphi_1$is the optical phase difference introduced between adjacent grating strips and J_n the nth order Bessel function of the first kind. Equation SI4 was used to calculate first-order diffraction efficiency for a 175 µm thick combined amplitude-phase grating as a function of absorbance and refractive index difference between alternating strips (Δn) at a wavelength of 490 nm. The resulting plot is provided in Figure SI3 and shows that for Δn = 0, diffraction efficiency of first diffracted order increases with absorbance. Furthermore, for Δn = 0, the maximum diffraction efficiency of first diffracted order value of is limited to 6.25%.

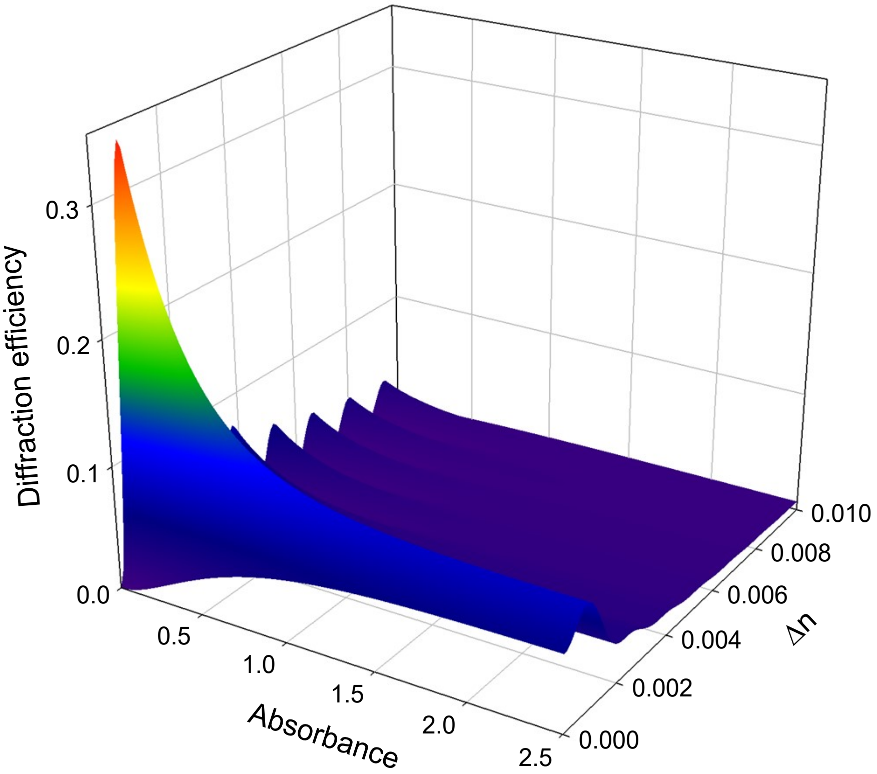

Figure SI3: Plot of first-order diffraction efficiency for a 175 µm thick combined amplitude-phase grating as a function of absorbance and refractive index difference at a wavelength of 490 nm

Differentiating Equation SI4 with respect to absorbance gives the sensitivity of diffraction efficiency to absorbance with the result given by Equation SI5:

$$\frac{\partial \eta_1}{\partial A_a} = \frac{\ln(10)\left(1 - 10^{-A_a}\right)}{8 \times 10^{A_a}}[J_0(\varphi_1) - J_2(\varphi_1)]^2 - \frac{\ln(10)\left(1 + 10^{-A_a}\right)}{2 \times 10^{A_a}}[J_1(\varphi_1)]^2$$

(SI5)

Equation SI5 was used to calculate the absolute sensitivity of the first-order diffraction efficiency to absorbance ($\partial\eta_1/\partial A_a$) as a function of both absorbance and (Δn) at a wavelength of 490 nm. The resulting contour plot (see Figure SI4) shows that $\partial\eta_1/\partial A_a$ decreases as absorbance increases.

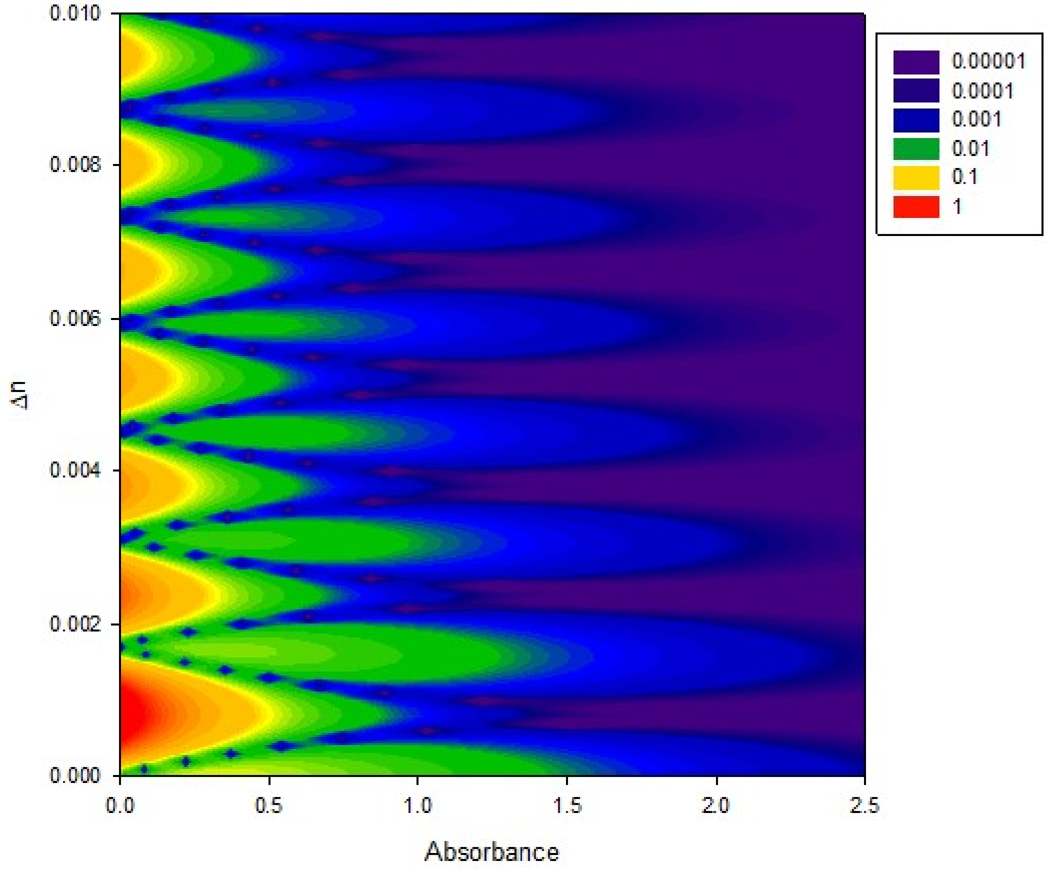

Figure SI4: Contour plot of the absolute sensitivity of the first order diffraction efficiency to changes in absorbance ($\partial\eta_1/\partial A_a$) as a function of absorbance and refractive index difference

We can determine maxima in $\partial\eta_1/\partial A_a$ by differentiating Equation SI5 again and finding zeros of the resulting expression (Equation SI6):

$$\frac{\partial^2 \eta_1}{\partial A_a^2} = \frac{\ln^2(10)\left(10^{A_a} - 2\right)}{8 \times 10^{2A_a}}[J_0(\varphi_1) - J_2(\varphi_1)]^2 + \frac{\ln^2(10)\left(10^{A_a} + 2\right)}{2 \times 10^{2A_a}}[J_1(\varphi_1)]^2$$

(SI6)

When $\Delta n = 0$, $[J_0(\varphi_1) - J_2(\varphi_1)]^2 = 1$ and $[J_1(\varphi_1)]^2 = 0$, so $\partial\eta_1/\partial A_a$ reaches a maximum when:

$$\frac{\ln^2(10)\left(10^{A_a} - 2\right)}{8 \times 10^{2A_a}} = 0 \tag{SI7}$$

Which occurs when $10^{A_a} = 2$ or alternatively when $A_a = \log(2) = 0.301$.

References

1. P. Berroy, M. L. Viriot and M. C. Carré, *Sensors and Actuators B*, 2001, **74**, 186.
2. B. Brycki, A. Szulc, H. Koenig, I. Kowalczyk, T. Pospieszny and S. Górka, *Comptes Rendus Chimie*, 2019, **22**, 386.
3. S. Abbott, CMC Values, https://www.stevenabbott.co.uk/practical-surfactants/cmc-values.php, (accessed 29/08/2021, 2021).
4. H. Kogelnik, *The Bell System Technical Journal*, 1969, **48**, 2909.
5. S. Li, R. A. Lessard and P. Galarneau, *Journal of Modern Optics*, 1990, **37**, 1319.
